# Control of automated behavior: insights from the discrete sequence production task

**DOI:** 10.3389/fnhum.2013.00082

**Published:** 2013-03-19

**Authors:** Elger L. Abrahamse, Marit F. L. Ruitenberg, Elian de Kleine, Willem B. Verwey

**Affiliations:** ^1^Department of Experimental Psychology, University of GhentGhent, Belgium; ^2^Department of Cognitive Psychology and Ergonomics, University of TwenteEnschede, Netherlands

**Keywords:** motor skill, sequence learning, automated behavior

## Abstract

Work with the discrete sequence production (DSP) task has provided a substantial literature on discrete sequencing skill over the last decades. The purpose of the current article is to provide a comprehensive overview of this literature and of the theoretical progress that it has prompted. We start with a description of the DSP task and the phenomena that are typically observed with it. Then we propose a cognitive model, the dual processor model (DPM), which explains performance of (skilled) discrete key-press sequences. Key features of this model are the distinction between a cognitive processor and a motor system (i.e., motor buffer and motor processor), the interplay between these two processing systems, and the possibility to execute familiar sequences in two different execution modes. We further discuss how this model relates to several related sequence skill research paradigms and models, and we outline outstanding questions for future research throughout the paper. We conclude by sketching a tentative neural implementation of the DPM.

## Introduction

Many of our daily activities are testimony to the possession of motor skill. One may think of riding a bike, lacing a shoe, or writing one's signature. Accordingly, within the fields of cognitive psychology and cognitive neuroscience ample research has been devoted to understanding how the brain represents and controls motor events. This venture is hindered, among other things, by a lack of direct conscious access to motor processes, and by the considerable time that the acquisition of motor skill typically takes. Nevertheless, various experimental tools have been developed over the last decades from which the workings of motor control—and its constant interaction with higher-order cognition—can be inferred with surprising detail. These experimental tools may be classified within two major experimental paradigms, motor adaptation^1^, and motor sequence learning (e.g., Doyon et al., 2003). The focus of the current paper is on motor sequence learning.

Motor sequence learning refers to the acquisition of the skill to rapidly and accurately produce a sequence of movements with limited effort and/or attentional monitoring. Such learning is typically based on repeated practice and (a mixture of) explicit instruction, explicit trial-and-error discovery and more elaborated hypothesis testing, or implicit detection of regularity. As most, if not all, of our goal-directed actions involve some kind of sequential structure, the human capacity to acquire sequential motor skill has been a topic of extensive research over the last decades. This research has led to a large variety of laboratory sequence acquisition tasks that typically involve finger-to-thumb opposition movements, finger presses on response boxes or key boards, movements of the whole arm, isometric forces, or oculomotor movements. The purpose of the current article is to provide a comprehensive overview on the contribution of one of these tasks, the *discrete sequence production* (DSP) task (Verwey, 2001), to our understanding of the execution of well-learned, discrete movement patterns.

The current review, then, is narrow in focus in the sense that it centers on work with the DSP task. Other sequence learning tasks and their major findings will not be discussed in detail (they have been reviewed elsewhere before: e.g., Rhodes et al., 2004; Perruchet and Pacton, 2006; Doyon et al., 2009; Abrahamse et al., 2010; Rosenbaum, 2010). However, the current review ultimately aims to outline from the DSP research a framework for sequence skill that aspires to a much broader application. This framework builds on the notion that sequential control occurs at both the cognitive level and at an autonomous motor level, and that it is the interplay between these levels that optimizes performance in sequential movement tasks.

In the next section we will (a) provide a description of the DSP task, (b) situate the DSP task within the larger domain of motor sequence learning in order to identify both its strengths and limitations, and (c) provide an overview of the typical phenomena associated with the DSP task. Overall, this section thus constitutes a sort of user's manual of the DSP task. In the third section, we will present the framework. This so-called *dual processor model* (DPM) was proposed already by Verwey (2001). However, based on more recent work with the DSP task, we here extent and specify the model. Finally, in the fourth section we will describe a tentative neuropsychological architecture that may underlie the DPM.

## The DSP task: a user's manual

### Experimental setting

**Figure 1:**
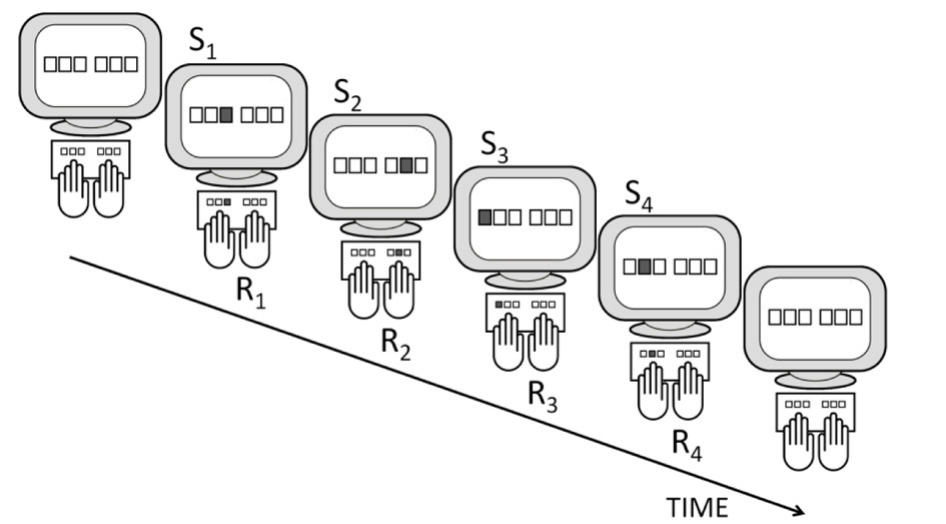
**A depiction of a typical DSP task including a 4-key sequence: responding (R_1_–R_4_) to a series of stimuli (S_1_–S_4_) with RSI = 0 ms**.

The DSP task involves participants resting four to eight fingers on the designated keys of the keyboard (Figure 1 and Table 1)^2^. A similar number of placeholders (usually small squares) is displayed on the screen, and each placeholder corresponds to one of the keys of the keyboard in a spatially compatible manner. Whenever a placeholder is lights up, the participant is instructed to rapidly press the spatially compatible key. Then the next stimulus is displayed. A typical DSP sequence involves two fixed series of 3–7 stimuli which results in the execution of two equally long key-press sequences. Usually, these sequences are carried out in a random order. This implies that a DSP task with, for example, two alternative 6-key sequences turns with practice from two series of 6-choice RT tasks into a single 2-choice RT task in which an entire 6-key sequence constitutes a single response. We use *S*_*n*_ to denote the *n*-th stimulus of a sequence, R_*n*_ to denote the *n*-th response in the sequence, and T_*n*_ to denote the RT associated with S_*n*_. Sometimes these RTs are referred to as inter-key-intervals (IKIs) but this only holds in the typical case when response-to-stimulus-intervals are 0 ms.

**Table 1:** **Standard settings of the typical DSP task**.

**Variable**	**Settings**
Effectors	2 hands (4/6/8 fingers)
Number of practiced sequences	2
Practice trials	500–1000 rep./sequence
Sequence length	3–8 stimuli/responses
Sequence structure/complexity	Arbitrary order: not based on pre-stored chunks or simple rule knowledge
Stimuli	Spatially compatible and key-specific

See the main text for elaboration.

Two methodological features of the DSP task are worth noting. First, the DSP task starts off with a practice phase (including 500–1000 repetitions per sequence) to develop the building blocks; These so-called *motor chunks* are assumed to represent a limited number of responses that can be selected and executed as if they are a single response in a control hierarchy (Book, 1908; Miller et al., 1960; Pew, 1966; Newell and Rosenbloom, 1981; Verwey, 1996). Following practice, the properties of these motor chunks are studied in a test phase in which a novel (“unfamiliar”) sequence is usually taken as control condition.

Second, by counterbalancing the fingers of individual participants across the sequential positions of the sequence, finger-specific effects at individual sequential positions are ruled out because each of the fingers contributes equally to the RTs at each sequential position. For example, when participants are using the D, F, G, J, K, and L keys on a keyboard, one participant may practice the 6-key sequence KFGDJL, the next participant the 6-key sequence LGJFKD (each key is shifted rightward relative to the first participant), and so on. This counterbalancing procedure also implies that the same sequences can be used as familiar and as unfamiliar, control, sequences so that RT differences between familiar and unfamiliar sequences are not related to inconspicuous differences in keying order, but rather are clean indicators of the underlying control processes.

### Situating the DSP task

We consider research with the DSP task as a way to study the building blocks of more complex behavioral patterns that make up everyday behavior (Paillard, 1960; Eysenck and Frith, 1977; Gallistel, 1980). For example, driving a car builds on movement sequences that underlie switching gears, steering through corners, looking in your mirror and back, etc. As such, the DSP task is representative for the way in which more complex real-world actions are acquired and controlled.

The DSP task was inspired by earlier studies that employed discrete keying sequences (e.g., Povel and Collard, 1982; Rosenbaum et al., 1983; Kornbrot, 1989). The use of key-press sequences to study the development and application of sequential skills has the benefit that they allow exploring sequential control *per se* because executing a single sequence element takes very little time (e.g., MacKay, 1982; Rhodes et al., 2004). This makes RTs in a keying sequence a more sensitive indicator for the underlying control processes as compared to when, for example, series of arm movements are studied and control processes may occur during execution of individual sequence elements (which will take relatively long).

Various other tasks have been used to study the acquisition and control of sequential movement skills, such as the pursuit rotor task (e.g., Grafton et al., 1992), the tracing of cut-out mazes (e.g., Van Mier et al., 1998), the *m* × *n* task (Hikosaka et al., 1995), a sequential elbow flexion and extension task (Park et al., 2004) and the serial reaction time (SRT) task (e.g., Nissen and Bullemer, 1987). Two of these tasks are especially interesting to elaborate upon here because their experimental designs overlap substantially with the DSP task; that is, they also aim at studying sequential representation on the basis of repeatedly performing key-press sequences. First, the *m* × *n* task involves trial-and-error based responding to sets of stimuli that eventually end up in fluent sequential skill. Like with the DSP task, the *m* × *n* task allows for exploring motor chunking; however, because practice involves trial-and-error search followed by relatively few repetitions once the sequence is fully discovered (i.e., with virtually error-free performance), the task differs from the DSP task that focuses on fast and effortless skill acquisition. Still, as will be elaborated on below, the model that Hikosaka et al. (1999) derived from mainly the *m* × *n* task has substantial conceptual overlap with the model that we propose below on the basis of DSP studies.

Second, in the SRT task participants cycle through a fixed and continuously repeating series of stimulus-response (S–R) events. The regularity between events is not explicitly conveyed to participants beforehand, and participants are often picking up on the regularity (as shown by performance measures) without being aware of it. Hence, in contrast to the DSP task, the SRT task mainly involves an implicit learning paradigm and does not employ discrete sequences. More importantly even, the SRT task does not typically involve motor chunking (Jiménez et al., 2011), Again, despite these differences, below we claim that various aspects of SRT skill overlap with DSP skill.

The DSP task as defined here (cf. Verwey, 2001) can also be distinguished from various earlier discrete sequence learning studies in three respects. First, the typical practice phase in DSP studies involves the execution of two sequences for around 500–1000 repetitions each. This results in performance that is characterized by substantial preparation before execution starts, which is indicated by the very fast RTs after T_1_ (sometimes reaching averages below 100 ms), and the alleged use of motor chunks. Earlier research employed much less practice. For example, Restle (1970), Simon (1972), Jones (1974), and Rosenbaum et al. (1983) employed only a few dozen repetitions per sequence. As it is known that the amount of practice has both quantitative and qualitative (e.g., differential sensitivity to interference from secondary tasks; e.g., Poldrack et al., 2005) effects on sequence skill, this might limit the generalizability of results from DSP studies to less practiced movement sequences. However, as we outline below, we believe that the framework we propose still has ramifications for situations with substantially less or more practice.

Second, the DSP task as defined here employs spatially defined key-specific stimuli that are presented throughout practice. These are mapped in a spatially compatible way to the response keys in order to minimize effects of (new) S-R learning. This differs from many earlier discrete sequence learning studies, in which participants were asked to explicitly learn the sequences after which their execution was triggered by either a simple go-signal (Rosenbaum et al., 1983, 1986) or by a pre-learned indicator (e.g., “O” for sequence 1 and “X” for sequence 2; Rosenbaum et al., 1984), or they were presented with word (or letter) series that were then to be spoken or typed in response to a go-signal (Sternberg et al., 1978).

Finally, the aim of DSP research is to explore the creation and exploitation of newly acquired sequence representations that ultimately lead to the development of motor chunks. It does not typically employ sequences that are described by pre-stored chunks or rule knowledge (like 12344321 and 12123434, Restle, 1970; Jones, 1981; Rosenbaum et al., 1983). In that situation, sequence learning is a matter of recognizing and reproducing the underlying rules rather than learning an arbitrary series of movements (cf. Coynel et al., 2010).

Hence, the DSP task as first specified in Verwey (2001) can be distinguished from earlier work on discrete sequence learning in terms of the overall amount of practice, the sequential structure, and the learning procedure. Later in this paper we return to these distinctions and elaborate on how we believe that they relate to the theoretical framework we propose. We will now first describe some of the major phenomena that are systematically observed across DSP studies.

### Typical phenomena

The literature on the DSP task reports a number of robust findings. These include (a) distinct phases of discrete sequence skill, and the spontaneous segmentation of longer sequences, (b) distinct coding systems that underlie sequence representations, and (c) the development of explicit sequence knowledge.

#### Processing phases of sequence skill: initiation, concatenation and execution

**Figure 2:**
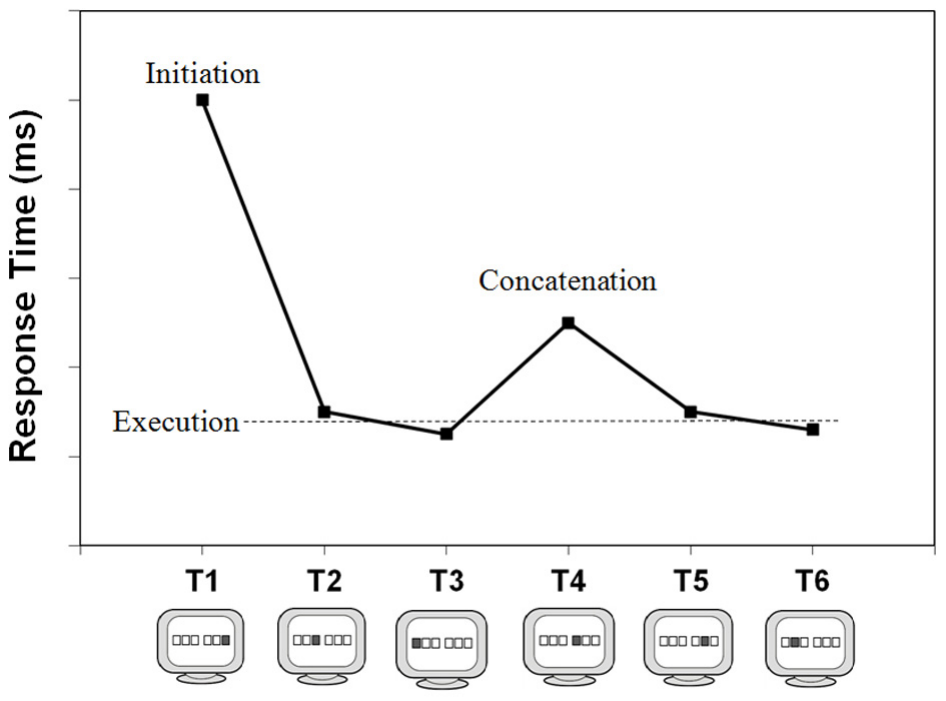
**Executing a 6-key sequence and its typical reaction time pattern.** It involves the processing phases initiation, concatenation, and (mere) execution. Please note that with smaller sequence lengths (<5 key-presses) the relatively slow T half way through (concatenation) is not typically observed.

The overall execution of a well-learned keying sequence can be related to three distinct processing phases that we believe are reflected in the respective RTs. The first phase is here referred to as sequence *initiation* and is reflected in T_1_. In case of a choice RT paradigm such as the typical DSP task, T_1_ is assumed to involve the selection and preparation of the sequence. As Figure 2 illustrates, this first key-press is typically much slower than subsequent key-presses (e.g., Verwey, 1999). This slow start is caused, in part, by suboptimal anticipation to the presentation of S_1_, as the slow first response can be observed even when a short, random series of key-presses is carried out (Verwey, 2003b). However, when there is a fixed keying order the difference between the first and later Ts increases considerably with practice because of the increasing possibility to prepare the later key-presses (Verwey et al., 2010). Possibly, the tendency to prepare an increasing number of elements also affects T_1_ itself: decreases of T_1_ with practice may be counteracted by the increasing time to prepare more responses in advance as the sequence becomes more familiar.

In line with the notion that T_1_ involves selection and preparation of forthcoming key-presses, T_1_ has been found to increase with the number of elements (i.e., key-presses) in the sequence (e.g., Verwey, 1999). This sequence length effect is commonly explained by the notion that individual responses are loaded immediately before sequence initiation into a short term *motor buffer* (Henry and Rogers, 1960; Sternberg et al., 1978; Hulstijn and Van Galen, 1983; Van Galen, 1991; Thomassen and Van Galen, 1992).

The sequence length effect appears to level off as sequences get longer (Sternberg et al., 1978, 1988; Rosenbaum, 1987). This is attributed to the notion that only a limited number of responses can be prepared in the motor buffer, and that preparation of later responses is postponed until after sequence initiation. This is referred to as concurrent, or on-line, programming. A related phenomenon is that the sequence length effect on T_1_ reduces with practice. This has been observed for, among others, the DSP task (Verwey, 1999). As the reduction of the sequence length effect with practice is associated with sequence-specific improvement (Verwey, 1999), it is assumed that this reduction indexes the development of a motor chunk that allows an entire sequence—or at least the first part of it—to be initiated like a single response.

The key-presses following sequence initiation are typically very fast—sometimes with RTs below 100 ms. This is possible because these involve just execution processes; selection and preparation processes of these keys have already occurred during the initiation phase. Together, these key-presses are referred to as the (mere) *execution* key-presses (see Figure 2). Key-presses reflecting initiation and execution can be dissociated through experimental manipulations. For example, Verwey (1999) showed that reversing the mapping between a sequence-specific stimulus and the sequence slowed initiation but not execution (see also Verwey et al., 2009).

Usually, longer sequences (>4 key-presses) show a relatively slow response half way through the sequence (Brown and Carr, 1989; Verwey et al., 2002; Kennerley et al., 2004; Bo and Seidler, 2009). Based on this observation, and the aforementioned finding that the sequence length effect levels off as sequence length increases, Verwey and Eikelboom (2003) argued that longer, fixed sequences are divided into multiple motor chunks due to assumed limitations in the length of a single motor chunk—in strong analogy to the well-known chunk-based capacity limitations of working memory (Miller, 1956; Cowan, 2000). Detailed examination of the effects of extensive practice and regularities in key-pressing order suggested that indeed most participants executed a 6-key sequence as 2 or more successive segments. Such segmentation is complemented by what is referred to as *concatenation*: the processes that allow distinct motor chunks within a sequence to be executed in rapid succession as smoothly as possible. The relatively slow response halfway through, then, is assumed to index the transition from one motor chunk to the next, and can be referred to as the concatenation point (see Figure 2). The slowing may be indicative of the involvement of higher cognitive processes such as preparation processes for the upcoming motor chunk (e.g., Verwey et al., 2010), or strategic parsing (Wymbs et al., 2012), and may eventually disappear with extensive practice when the initially separated motor chunks become rearranged and behave as a single larger motor chunk.

The idea that concatenation involves other processes than mere execution of key-presses is supported by a double dissociation between execution and concatenation key-presses; they have been shown to be affected by different manipulations. Specifically, the RTs reflecting the concatenation point increased less than RTs from execution key-presses after changing the location of the hand relative to the body (De Kleine and Verwey, 2009a), when using fingers adjacent to the ones used during practice (Verwey et al., 2009), and when discrete sequences were executed by dyslexics (De Kleine and Verwey, 2009b). Conversely, the concatenation point was lengthened more than the execution key-presses after applying transcranial magnetic stimulation (TMS) to the pre-supplementary motor area (pre-SMA; Kennerley et al., 2004). Initiation and concatenation are assumed to both involve loading and initiating the upcoming motor chunk, but the initiation phase will most likely include more general preparatory processes too (Verwey, 2003b).

Various studies have explored the notion that higher cognitive processes are mainly involved in the concatenation of successive motor chunks. If so, a cognitively demanding secondary task should especially slow concatenation as compared to execution key-presses. After some initial contradicting findings (Brown and Carr, 1989; Verwey, 2003b), we recently explored this prediction with a secondary task that required participants to count tones that were presented at a random moment during sequence execution (Verwey et al., 2010, 2013). This secondary task indeed slowed responses, but slowing was not larger for the alleged concatenation response than for the other responses. This finding was explained by the notion that concatenating motor chunks in a fixed sequence does not necessarily require cognitive processing after substantial practice. Apparently, motor chunks can become associated within a single sequence representation, so that executing one motor chunk primes the commonly ensuing next chunk (just like individual responses can become associated in an SRT task, Abrahamse et al., 2010). This can explain why concatenation has been found to get faster with practice (e.g., De Kleine and Verwey, 2009a).

Overall, we thus propose that initiating, concatenating and executing key-presses involve distinct processes of sequence skill that are reflected in their respective RTs. This suggests that these distinct phases are differentially affected by various experimental manipulations.

***Imposing segmentation.*** For experimental purposes, it is a challenge that (depending on the structure of the sequence) the relatively long RT that is assumed to index the concatenation phase has been found to occur at different sequential locations for different persons. Consequently, across a group of participants a single long RT cannot always be easily observed (Sakai et al., 2003; Verwey, 2003b; Verwey and Eikelboom, 2003; Kennerley et al., 2004; Bo and Seidler, 2009). Instead, the second and the last responses are often faster than the responses in between (Verwey, 2003b; Verwey and Eikelboom, 2003). This could be interpreted as concatenation processes being distributed across these in-between responses for a group of individuals.

In the literature, several methods have been proposed for artificially imposing segmentation at the same location within the sequence across participants. A first procedure is to introduce regularities in response order. Such regularities appear to induce the same segmentation across participants (e.g., Restle, 1970; Povel and Collard, 1982; Koch and Hoffmann, 2000; Sakai et al., 2004). For example, De Kleine and Verwey (2009a) observed a highly similar segmentation across participants with their sequences, which was attributed to the occurrence of a reversal (A-B-A) halfway through the sequence. This particular regularity may have initially affected the parsing into subsets of responses, which eventually consolidated into motor chunks.

Second, when during practice a pause is inserted between two successive stimuli (yielding a so-called *prestructured* sequence), participants are typically observed to segment the sequence at the location of the pause when subsequently the pause is removed. This suggests that the position where concatenation occurs is determined by the pause position during practice (e.g., Stadler, 1993; Verwey and Dronkert, 1996; Verwey et al., 2009, 2010). The possibility that this segmentation involves learning of a particular temporal pattern, a rhythm, has been refuted because (a) the various intervals did not adhere to the expected integer ratios (Verwey, 1996; Verwey and Dronkert, 1996), (b) the temporal pattern did not transfer to another sequence (Verwey et al., 2009), and (c) segmentation patterns did not correlate with the individual's temporal control abilities (Bo et al., 2009; Bo and Seidler, 2009; also see, Sakai et al., 2004).

Finally, Jiménez et al. (2011) used differently colored key-specific stimuli to distinguish different segments in an SRT task (i.e., stimuli signaling the responses that were to be segmented together were presented in the same color). This successfully induced consistent segmentation/concatenation across participants, but has yet to be tested and validated for discrete movement sequences.

***Assessing segmentation and concatenation.*** Several methods have been reported to identify spontaneous chunking behavior in a *post-hoc* fashion. First, some studies have compared the slowest T after the T_1_ (assumed to be the concatenation point) against the others (e.g., Verwey et al., 2010). This procedure can be refined by first testing all T's (after T_1_) against its directly surrounding neighbors, and look for a significantly longer T that can subsequently be labeled as the concatenation point. However, this method relies on assumptions that during training chunk boundaries are relatively static and that, eventually, short chunks are not combined into larger chunks. This method is relatively insensitive to measuring how the chunking structures develop with practice.

Second, Jiménez et al. (2011) proposed a different manner of studying motor chunking. Instead of identifying the precise concatenation point, these authors developed a method to index chunk formation that was inspired by the logic of the analysis of variance. In brief, segmentation and concatenation of motor chunks are assumed to be indexed by an increase of the ratio between the variance between elements of the sequence and the variance within sequence elements. Hence, it relies on the variance concerned with differences in responding to distinct parts of the sequence (between-element variance), while controlling for variance caused by general factor such as practice or fatigue (within-element variance). It needs to be said, though, that this method was validated within the context of an SRT task, and has yet to be tested for a DSP task.

Third, Wymbs et al. (2012) modeled chunking behavior by using so-called modularity-optimization algorithms to seek for groups of T's (i.e., IKIs) that are more tightly connected to each other relative to their connections to T's in other groups. Such modeling allowed calculating a measure for the ease with which the network could be divided into smaller communities, and the inverse of this measure was used to index chunk magnitude. This procedure allows tracing chunk development over practice.

#### Coding movement sequences

Several studies have investigated the type of representation that forms with practice in discrete movement sequences. The general notion is that initial sequence execution relies on effector-unspecific sequence knowledge (also referred to as effector-independent coding) and that with practice execution becomes increasingly dependent on effector-specific knowledge (also referred to as effector-dependent coding; Hikosaka et al., 1999; Bapi et al., 2000; Verwey, 2001; Verwey and Wright, 2004; Verwey et al., 2009).

Verwey and Wright (2004) examined the contribution of effector-dependent and -independent representations with respect to sequence learning in the DSP task. In their study, participants practiced two 5-key sequences, using three fingers of either a single hand or across both hands. When performing these sequences with the unpracticed hand configuration in a subsequent test phase, execution was slower than with the practiced hand configuration. Still, it was faster than the execution of unfamiliar sequences. This finding suggested that with extensive practice in the DSP task the sequence representation includes an effector-dependent and an effector-independent component.

In a subsequent DSP study, Verwey et al. (2009) found that the execution rate of 6-key sequences was slowed also when participants used the adjacent fingers of the same, practiced hands. However, this slowing was clearly less than in Verwey and Wright's (2004) study in which transfer to fingers of the other hand was assessed. The authors suggested that effector-specificity in the DSP task may result from hand-based visuo-spatial coding: using adjacent fingers could well allow involvement of the same hand-based reference frame for coding locations as during practice (e.g., Cho and Proctor, 2002). That hand-based spatial coding is probably not the whole story, however, is suggested by indications that effector-specific sequence learning involves adjustment to the biomechanical properties of the effector used (Park and Shea, 2003), and that one effector may start moving before the previous movement has been executed (i.e., co-articulation; Daniloff and Moll, 1968; Jordan, 1995; Sosnik et al., 2004; Berner and Hoffmann, 2009).

Finally, the extent to which sequence coding involves effector-dependent and -independent information may be related to the experimental design too, as indicated by the following discrete sequence studies: (a) Bapi et al. (2000) showed that with practice reliance on an effector-independent representation decreases, and control becomes more effector-specific (i.e., motor based; cf. Hikosaka et al., 1999; Park and Shea, 2003). (b) Gruetzmacher et al. (2011) showed that only physical but not observational practice results in coding in motor coordinates. (c) Several studies showed that with extensive practice, representations for key-pressing sequences include an effector-dependent component (e.g., Bapi et al., 2000; Verwey and Wright, 2004; Verwey et al., 2009), while for elbow flexion and extensions sequences effector-independent representations seem to remain dominant with extended practice (Kovacs et al., 2009b). (d) The complexity of a movement sequence influences the use of motor as opposed to visuo-spatial representations (Kovacs et al., 2009a; Panzer et al., 2009). Finally, (e) Panzer et al. (2011) suggested that the coding of movement sequences depends on individual characteristics in that with a relatively complex flexion/extension sequence older participants (over 60) appeared to rely more on motor coding while young adults (23–31 years) used visuo-spatial coding.

In sum, there is now substantial reason to believe that sequential movement skill involves several types of representation. Some involve a slowly developing motor code (e.g., in terms of joint angles and forces), while other representations probably code movement patterns in terms of more rapidly developing spatial reference systems (Hikosaka et al., 1999; Panzer et al., 2009). Finally, even abstract symbolic codes, like verbal codes, may be used. Which codes are dominant in a particular task seems to depend on the amount and type of practice, the number and type of responses in the sequence, individual capacities, and the strategies used during practice.

#### Explicit sequence knowledge

It is usually accepted that sequence learning can be both implicit and explicit. Implicit learning refers to a learning process that proceeds in the absence of conscious awareness of both the learning itself and the end product of learning. As mentioned above, implicit learning is the main object of study in the SRT literature. Explicit knowledge may be based on explicit sequence descriptions in the instructions, but can also develop online by testing hypotheses about the regularity of events (e.g., Haider and Frensch, 2005; Rünger and Frensch, 2010).

Participants in DSP studies are commonly informed that they are performing fixed keying sequences. In combination with the saliency of DSP sequences this has led to the notion that the DSP task is an explicit sequence learning paradigm (Bo and Seidler, 2009). However, it has been demonstrated that participants in DSP studies do not always possess explicit, in-depth and verbalizable knowledge of the order in which the elements were carried out (e.g., Verwey et al., 2010). That is, they have no *structural knowledge* even though they know that there is a fixed regularity in the sequences (i.e., *judgment knowledge*, Dienes and Scott, 2005). Furthermore, even when participants were able after the experiment to report on the structure of their sequences, a substantial number of them indicated to have reconstructed this knowledge in the recall task after the experiment by tapping the sequences in their mind or on the table top (e.g., Verwey et al., 2010; Verwey and Abrahamse, 2012). Two potential explanations may underlie this lack of explicit, structural knowledge of the DSP sequences. It may be that participants obtain substantial (or full) explicit knowledge of the sequential structure early on in training, but later gradually lose out on it as performance becomes more and more automatized. Alternatively, some participants may never develop structural sequence knowledge. Interestingly, participants with substantial structural knowledge are often only a little faster than less aware participants—if any. This indicates that skill in this task does not depend much on explicit (structural) knowledge (Verwey et al., 2009, 2010; Verwey, 2010), in line with the notion that in the DSP task motor coding is dominant.

Here we finish the user's manual of the DSP task. In the next sections we will first describe a framework on discrete sequence skill referred to as the DPMDPM that we have derived from our work with the DSP task, and then provide a tentative neuropsychological architecture that may underlie the DPM.

## Cognitive underpinnings of discrete sequence execution

Over the last decades, various cognitive models have been proposed to account for our capacity to develop sequential skill. Here we present an updated version of the DPM, which has resulted from work with the DSP task. Additionally, we speculate about its relationship with sequencing models that have been developed in different research paradigms.

### Dual processor model

The DPM claims that a cognitive processor and a motor processor are responsible for skill in executing discrete movement sequences. During early practice, the cognitive processor translates each externally presented stimulus into the associated response, and prompts the motor processor to execute this response. In case of relatively novel but explicitly known sequences (e.g., through instructions), it may also load, one by one and before execution, a limited number of individual responses into the motor buffer. This motor buffer is assumed to be a part of working memory (Smyth and Pendleton, 1989; Tattersall and Broadbent, 1991; Verwey, 1999). However, as short series of movements are repeatedly executed in close temporal proximity, these series are assumed to gradually integrate into a single representation, the motor chunk. The availability of motor chunks allows the cognitive processor to eventually select and load this motor chunk from long term memory in a single processing step into the motor buffer, as if each motor chunk constitutes a single response (Verwey, 1999).

After loading the motor buffer, the cognitive processor triggers the motor processor to start reading the codes for the individual movements from the motor buffer and to execute the movement series in a relatively autonomous fashion. The rapidity with which familiar sequences can be selected and executed through this buffer-mediated process, is what makes up the sequence skill. According to the DPM sequential movement skills can be considered automatic to the extent that (a) little cognitive processor involvement is required when motor chunks are executed by the relatively autonomous motor processor, and that (b) with practice the contribution of the cognitive processor may even be further reduced as entire motor chunks may become triggered by external stimuli as if they involve prepared reflexes (cf. Hommel, 2000).

The model has two additional features. First, when the task, participant strategy and the available processing resources allow it, the cognitive and the motor processor may “race” each other to initiate each response in a familiar sequence; the motor processor triggers the individual responses stored in the motor buffer, while the cognitive processor selects each response on basis of key-specific stimuli (Verwey, 2001) or by using explicit sequence knowledge (Ruitenberg et al., 2012). This race will be elaborated upon below.

Second, whereas the cognitive processor initially is responsible for selecting each motor chunk and loading it into the motor buffer, with practice this may automatize for the later motor chunks of a sequence. That is, associations between successive motor chunks—in strong analogy with associative learning between individual responses in, for example, the SRT task—may facilitate or even take over the selection and loading (i.e., the concatenation) processes from the cognitive processor. Empirical support for this notion was provided by Verwey et al. (2010, 2013), who showed that the concatenation interval is not slowed any more by a secondary task than other key-presses. This suggests that, after substantial practice, the cognitive processor is no longer required for concatenating motor chunks when they are repeatedly executed in a fixed order.

#### Dual processors

Two major issues for the DPM concern the justification for the assumptions of (a) two distinct processors instead of a single graded processing resource, and (b) a race between the two processors. We believe that justification for the two processor assumption comes from several findings. The first relates to the notion that action slips have been found to mainly occur at the decision points in an action sequence, where higher-cognitive involvement is required for adequate action selection (e.g., Reason, 1992; Botvinick and Bylsma, 2005), and not the moments where behavior is guided more automatically. This is in line with two qualitatively distinct processors; one controlling and the other executing behavior. Similarly, two such processors can also explain why action sequences sometimes continue even though the situation requires sudden termination. In that case the cognitive processor is temporarily unavailable (e.g., by distraction) or disengaged (e.g., in case of absent-mindedness), and the motor processor simply continues the habitual course of action. Second, we believe that two distinct processors fit well with the notion that both the qualitative features and underlying neural substrate differ greatly between early and late practice stages. Below this is discussed in more detail.

Third, and most importantly, there is also empirical support for two processors from DSP studies. One source of support is that selecting a forthcoming action (a single key-press, or a motor chunk) slows ongoing sequence execution, but this slowing is unaffected by the load of the selection process itself (when manipulated in terms of S-R compatibility and reversing a learned stimulus-sequence association, Verwey, 1995, 2001). This cannot be easily explained by a single resource or single processor model. Another type of behavioral support comes from a dual task study by Verwey et al. (2010). This study involved a tone counting task as secondary task to force participants to allocate their cognitive processor away from executing the sequence (for an earlier version, see Verwey, 1993). It appeared that in familiar sequences each tone was followed by slowing of the three ensuing responses by maximally 30 ms. In a follow-up study, Verwey et al. (2013) further showed that slowing was larger for identifying and counting a tone than for merely identifying a tone. These dual task findings are in line with two processors: while the secondary task allocated the cognitive processor away from executing the sequence, the motor processor enabled the continuation of sequence execution—with the moderate slowing being caused by the cognitive processor no longer racing with the motor processor. Additionally, taking away the key-specific stimuli (after the first) in a familiar keying sequence has been found to also slightly slow sequence execution (Verwey, 1999, 2010). This is entirely consistent with the notion that this largely eliminated the contribution of the cognitive processor to triggering individual responses in the familiar keying sequence—with performance based merely on efforts of the motor processor.

We would like to close this section by outlining how the DPM rests on assumptions similar to models developed for various other types of tasks. First, the notion of separate cognitive and motor processors is found across (models derived from) various research paradigms. For example, Sternberg (1998) suggested that sensory and motor processing stages might be carried out by processors independent from a central processor that is responsible for cognitive processing stages (like stimulus identification, and response selection). Moreover, results obtained with the Psychological Refractory Period (PRP) paradigm (e.g., Welford, 1952; Pashler, 1994) showed that the processing stages that are affected by a central bottleneck include response selection, response initiation, decision, and certain perceptual judgments (e.g., Pashler, 1992, 1994; De Jong, 1993). While the central bottleneck may be caused by a cognitive processor dealing with one process at the time, the initial perceptual processes and the final motor execution stages are assumed to be carried out by dedicated processors (Pashler, 1994). Indeed, the overall notion that a cognitive processor performs a prepared series of processing operations has been proposed many times before in more general information processing architectures (e.g., Norman and Shallice, 1986; Detweiler and Schneider, 1991; Meyer and Kieras, 1997; Anderson et al., 2004; Salvucci and Taatgen, 2008). The order of these processing stages, and whether sensory and motor processors are to be used, would be set during task preparation by creating a superordinate control structure (e.g., Norman and Shallice, 1986; De Jong, 1995; Klapp, 1995; Salvucci and Taatgen, 2008). Such a schema-based processing procedure is in line with our notion of a cognitive processor setting in advance the processing operations and autonomous processors to be used.

Second, the notion that different processors are racing to trigger a response in a familiar keying sequence fits well with the many indications that the execution of a movement sequence involves the simultaneous use of different codings (motor, egocentric, and allocentric spatial, verbal; see e.g., Hikosaka et al., 1999; Bapi et al., 2000; De Kleine and Verwey, 2009a; Verwey et al., 2010; Panzer et al., 2011; Shea et al., 2011; Verwey and Abrahamse, 2012). Moreover, it relates strongly to other models that assume a race between different processing routes (e.g., Logan, 1988; Kornblum et al., 1990).

#### Modes of sequence execution

Verwey (2003a) noted that sequencing performance in the DSP task can be based on at least two execution modes. The first is a *reaction mode* in which participants use each key-specific stimulus to select a response. This mode is especially used when encountering new sequences, and involves closed-loop control. As a discrete sequence is repeatedly executed, participants learn the order of stimuli and responses, and switch to performing the sequence (or short parts of it; i.e., motor chunks) in response to just the first stimulus. Subsequent stimuli can be ignored and participants are said to be performing in the *chunking mode*. This mode can be envisaged as open-loop control in the sense that key-specific stimuli after the first are no longer needed (though, as said, they may still be used when the cognitive processor races with the motor processor).

Recently, indications have been found that discrete keying sequences can be carried out in a third execution mode too. Earlier studies had demonstrated that when participants switch from slow to fast execution of a familiar sequence they briefly produce the sequence at some intermediate rate (Verwey, 2003a), and that elderly do not use motor chunks in discrete keying sequences but still benefit from practice (Verwey, 2010; Verwey et al., 2011). Inspired by these findings, Verwey and Abrahamse (2012) tested the notion that an SRT-like *associative mode* develops with DSP practice. In this mode successive reactions are primed by the preceding responses but still require stimulus processing for actual execution—as would occur in SRT learning (see Abrahamse et al., 2010). Verwey and Abrahamse (2012) argued and confirmed that in the DSP task the effect of the associative mode would emerge only when the much faster chunking mode is not used. Skilled participants performed a condition in which familiar, discrete keying sequences were carried out while most of them included 2 deviants (i.e., key-specific stimuli at unpredictable positions) that effectively disabled the chunking mode. As expected, the few sequences in this condition without deviants were executed much slower than the familiar sequences in a non-manipulated condition. Importantly, however, they were executed faster than unfamiliar sequences. Analysis of the RT distributions showed that this effect could not be attributed to sequences occasionally being performed in the chunking mode. The authors interpreted the intermediate execution rate as resulting from reactions to stimuli being primed by the preceding responses, just as observed by Verwey (2003a). That this associative mode develops seems reasonable given that responding to successive stimuli in early DSP practice mimics the SRT task.

These findings led to the proposal that familiar movement sequences can be executed in two different modes, the associative mode which continues to require external guidance by movement-specific stimuli and does not involve no use of motor chunks, and the chunking mode which is based on advance preparation of motor chunks and which does not require guidance by movement-specific stimuli. In the next section we attempt to integrate these execution modes with the DPM.

#### A general architecture

The reaction and chunking modes can be easily accounted for by the DPM (see below). The theoretical challenges concern the implementation of the associative mode, especially with respect to the representational level. It is generally accepted that representing sequential information may involve coding across the perceptual, cognitive, and response-based/motor levels (e.g., Hikosaka et al., 1999; Keele et al., 2003; Abrahamse et al., 2010; Goschke and Bolte, 2012). The chunking mode would mostly depend on associations at the motor level from which motor chunks can develop. Conversely, the associative mode could be tentatively linked to various types of visuo-spatial associations—in line with the SRT literature (Abrahamse et al., 2010). However, the possibility cannot be excluded that the associative mode derives directly from the same associations that underlie the chunking mode: rather than being just static propositions waiting to be used for the chunking mode, motor chunks may continuously influence ongoing processing (Cleeremans, 2008). They may, for example, prime the selection of individual responses. To comply with the notion of distributed coding (cf. Hikosaka et al., 1999; Abrahamse et al., 2010), we assume an event-based sequence representation—where *event* refers to a specific S-R episode—that potentially involves associations at both the visuo-spatial (e.g., between successive stimuli or response locations) and motor level. Its precise features will probably depend on the task requirements, the context, and the amount of practice.

**Figure 3:**
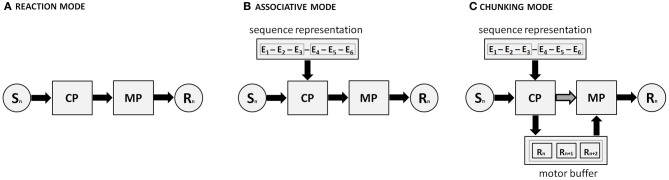
**The dual processor model (DPM) involves a cognitive processor (CP) and a motor processor (MP) that together drive three distinct modes of sequence execution, through long-term sequential knowledge and the temporary storage in a motor buffer (in the case of motor chunking).** S_*n*_ and R_*n*_ denote the current stimulus and corresponding response within the sequence, respectively. Black arrows and boxes denote the relevant processing routes. **(A)** In the reaction mode, responses are selected by the cognitive processor (CP) on the basis of S-R translation. **(B)** Ongoing response selection by the CP is facilitated by the first, still weak, sequence knowledge that develops. **(C)** Motor chunks have developed, and the CP selects these motor chunks, loads them in the motor buffer, from where the motor processor can execute them. Please note (I) that panel C also depicts the assumption of DPM that there can be a race between two response processes: the triggering of responses by the motor processor reading response related codes from the motor buffer, and response selection by the cognitive processor on basis of continued S-R translation or explicit sequence knowledge (dark gray arrow with black lining). Also note (II) that a fourth theoretical possibility is not depicted here, namely that the CP can load the motor buffer *not* by selecting motor chunks, but *rather* by (the slower process of) selecting and loading individual response elements of a (relatively) unfamiliar sequence.

Figure 3 depicts a cognitive architecture for the skilled production of movement sequences. It shows how a response (R_*n*_) is generated on the basis of stimulus input (S_*n*_) by the concerted action of the cognitive and motor processors. These processors may use a motor buffer that can temporarily hold representations that concern a limited number of responses. In the reaction mode, which is dominant with unfamiliar or random sequences, the cognitive processor processes sensory input and selects the appropriate response separately for each particular stimulus. Next, it puts the motor processor to work for the actual execution of the response. With repeated execution of the same sequence of events, associations develop between successive events. The resulting representation allows for response selection processes to be primed when they are executed in a familiar order on the basis of preceding events (associative mode). Moreover, when the representation becomes sufficiently strong at the motor level, it allows for the temporary activation of a short series of movements (i.e., motor chunks) as if they are loaded in a single step into a motor buffer. Next, the motor buffer content is read and executed by the motor processor. Because the motor buffer capacity is limited, the number of simultaneously prepared and executed responses is limited. Finally, the independence of the cognitive processor and motor processor allows a race between them in that the cognitive processor selects responses at the cognitive (“response selection”) level, and the motor processor triggers responses from the motor buffer.

The DPM forwards a number of testable predictions on the dynamic interplay between the different modes and the types of sequence knowledge acquired. For example, the model predicts that for participants without explicit sequence knowledge, the effect of a secondary task on executing a DSP sequence will vanish if key-specific stimuli after the first are no longer presented (i.e., single-stimulus condition). The reason is that without explicit knowledge and external stimuli, the cognitive processor is no longer able to race with the motor processor, and thus never enhances skilled (i.e., motor processor based) performance. Additionally, if after extensive practice the chunking mode is prevented through, for example, introducing (auditory) stop-signals during a specific proportion of sequences within a block (requiring to terminate sequence execution), it can be expected that executing a familiar sequence in a single-stimulus condition is only better than executing an unfamiliar sequence for aware (and not for unaware) participants because their explicit knowledge still allows the cognitive processor to enhance performance beyond pure S-R translation. Furthermore, artificially slowing execution rate by using more complex responses will increase the presence, and contribution, of explicit sequence knowledge and/or the associative mode because there is more time to contribute. These and other (types of) predictions need to be addressed in future research.

### Generalizing the dual processor model

In our efforts above to situate the DSP task within the larger domain of sequence learning, we already anticipated a discussion about how the DPM relates to other work on sequence skill. Here we outline such a link, first, with respect to discrete sequence skill, and second, with respect to the models that stem from related sequence learning paradigms. This results in various issues for future research.

#### Practice levels and sequence complexity

The end-product of motor learning is typically related to automaticity in the sense that control over behavior becomes fully encapsulated and cognitively impenetrable. For example, it is difficult to verbalize the procedure of how one laces one's shoes. Without disclaiming this notion of automaticity in discrete sequence skill, the DPM features both cognitive and motor control as continuously interacting components of even well-trained movement sequences. This model is based on research with the DSP task, which typically employs sufficient practice to reach substantial performance improvements as compared to unfamiliar movement sequences but it does *not* account for overlearned sequences (such as when a single sequence is practiced for many sessions across multiple days or even weeks; e.g., Lehéricy et al., 2005; Coynel et al., 2010). Hence, the DPM may not generalize to overlearned movement sequences. However, we believe that overlearned sequence skill can still be explained by the DPM by assuming that with more extensive practice with the same movement sequence, the contribution of the cognitive processor is increasingly reduced as processing becomes automatized (i.e., stimulus-based selection of entire motor chunks; successive motor chunks becoming either fully represented into a larger motor chunk, or concatenated in a largely automatic manner).

As mentioned above, there are numerous earlier discrete sequence learning studies that employed relatively little practice, mostly in combination with a learning procedure that did not involve key-specific stimuli (Restle, 1970; Simon, 1972; Jones, 1974; Rosenbaum et al., 1983). We believe that these studies did not involve sequence execution in the chunking mode. Rather, performance in those studies seems to have been based on a dominant cognitive processor using simple rules that describe the entire sequence. As such, the phenomena observed in those studies seem to inform us primarily on the cognitive constraints of the cognitive processor.

One such major phenomenon that has been shown across multiple sequential motor tasks is referred to as the parameter remapping effect (Rosenbaum et al., 1986). This implies that a sequence is more difficult to execute when the number-of-taps carried out by a particular finger changes throughout the sequence than when each finger always taps a fixed number of times. One could say that the sequential structure provides limitations on the ease with which movement sequences are prepared. It is, however, not clear whether this effect can be found also after more substantial practice. The DPM suggests that the development of motor chunks could shield against interference by parameter remapping, but this requires explicit examination.

Finally, as noted above, the various discrete sequence studies that employed little practice *also* employed sequences of limited length and/or salient rule-based structure, which can be easily transferred to long-term memory with even little practice. This leaves two possibilities. First, it may be that the fast development of long-term memory representations for these short and/or rule-based sequences actually allows for motor chunking even with little practice. This is tentatively supported by the observation that practice on 3-key sequences quickly reaches a performance asymptote (e.g., Rosenbaum et al., 1983). Alternatively, motor chunking may be highly dependent on substantial practice, and involve different processing mechanisms (and neural correlates) than the execution of short and/or salient sequences with little practice. We here argue for the latter case, which is supported by the general notion that coding in motor coordinates requires ample physical practice, and the finding that the relatively high execution rate of simple 2-key sequences disappeared with increasing cognitive load (Verwey, 2001). As such, we believe that discrete sequence learning studies with short and/or rule-based sequences, too, are strongly based on a dominant cognitive processor that controls performance by the one-by-one loading of individual response elements with no motor chunks involved. Future studies are required to further explore this issue.

In short, even though the DPM is built on DSP studies that are characterized by substantial practice with relatively short, complex sequences, other discrete sequence learning studies can be tentatively related to this model, and—more importantly—can inform us about the characteristics of the two processors and their interplay.

#### Relationship with other sequence skill models

As mentioned above, the production of movement sequences has been studied with several tasks. The cognitive models that are proposed to account for the results in those studies share several features with the DPM. First and foremost, it should be noted that these models generally agree with the DPM that cognitive and motor processing involve independent systems (e.g., Pew, 1966; Allport, 1980; MacKay, 1982; Schmidt, 1988). One particularly interesting model has been proposed by Klapp (1995, 2003). He developed it for series of timed (Morse code) key-presses and speech sequences. It assumes, like the DPM, that longer sequences involve several chunks, each of which may initially consist of a single element (key-press or syllable) and later, of short series of these elements. The so-called INT process programs the internal structure of each chunk, which may in simple RT conditions occur before sequence initiation. After loading the motor buffer, and after the go-signal has been detected, the SEQ process then places these chunks in the correct order so that the sequence of chunks can be executed correctly. In longer sequences, the INT processes dedicated to later chunks occur during sequence execution (Klapp, 2003). One could argue that these INT and SEQ processes are a specification of two roles carried out by the cognitive processor proposed in the DPM when timing is crucial. Indeed, this model leaves actual execution to some unspecified motor process.

The Hikosaka et al. (1999) model suggests that, in what they called the pre-learning stage, each stimulus triggers a movement without any effect of preceding or subsequent stimuli (like the DPM's reaction mode). With practice, visuo-spatial and motor learning develop, with the former developing at faster rate. The visuo-spatial learning may be tentatively related to the associative mode of the DPM: successive events prime each other on the basis of visuo-spatial sequential representations, either at the perceptual (e.g., stimulus location learning) or the response (e.g., response location learning) level. The motor learning system becomes dominant during later stages of sequence learning, and can be tentatively linked to the chunking mode of the DPM.

Keele et al. (2003) proposed a dual system framework for sequence learning in the SRT task. This model is designed to explain results from a continuous sequence learning task that does not include preparation and chunk development. Instead, the main focus is on the implicit-explicit divide. The framework assumes a unidimensional system that is composed of multiple modules that each associate information within a single informational dimension. There also is a more overarching multidimensional system that enables associations both within and across informational dimensions. Together, these two systems can account for a number of dual-task studies on SRT learning. The DPM's cognitive processor is clearly reminiscent of Keele et al.'s (2003) multidimensional processor, but the unidimensional modules do not seem to correspond well to the motor processor of the DPM. Though the latter two share features in terms of their relatively autonomous functioning, there are some essential differences. Most importantly, whereas the motor processor is assumed to be executive in nature and fully dependent on input from the cognitive processor, the unidimensional modules from Keele et al. are primarily representational systems. Both the multidimensional system and the unidimensional modules are related to what we referred to as the associative mode: they are both responsible for the relatively automatic priming of responses on the basis of inter-trial contingencies and do not involve the possibility of preparing series of responses and using motor chunks. This is entirely reasonable given that the Keele et al. model was developed in the SRT research domain where motor chunks do not develop (e.g., Jiménez et al., 2011).

Finally, based on a number of discrete sequence learning studies with relatively little practice, Rosenbaum et al. (1984) and Rosenbaum (1987) proposed the hierarchical editor (HED) model. The HED model builds on the notion that a hierarchically organized motor program is first “edited” to specify open parameters, only after which the sequence can be executed. We believe, in line with notions from above, that the HED model mainly describes the cognitive constraints that are related to the workings of the cognitive processor in preparing and/or controlling sequence execution after limited practice. With substantial practice and the resulting development of strong motor chunks it may be questioned if similar hierarchical structures work on series of whole motor chunks.

Overall, we believe that there is a clear overlap between the DPM and these other models. This overlap supports the merit of the DPM as a general model of sequence performance. The most important features of the DPM are that (a) it distinguishes the associative and chunking modes of sequence execution (and thereby their respective literatures), (b) it is able to explicitly account for automaticity of skill by the relative autonomous execution processes of a motor system (motor processor and motor buffer), and (c) it allows for explaining the overall dynamic interplay between cognitive and automatic processes in daily life.

## Neural underpinnings of the dual processor model

In this section we discuss on the basis of cognitive-neuroscientific findings (e.g., Hikosaka et al., 1999; Ashby et al., 2010; Stocco et al., 2010; Penhune and Steele, 2012) how the cognitive architecture proposed above may be implemented in the human brain. Specifically, we develop a mapping of the DPM on specific cortico-striatal loops (Seger, 2006; Doyon et al., 2009; Ashby et al., 2010). The nature of this mapping is admittedly speculative as very little of the work discussed here strictly builds from the DSP task itself, but we feel that this effort nevertheless will inspire progress in the understanding of discrete sequence skill from a combined cognitive and neuroscientific approach.

We explicitly distinguish the three modes in which sequences can be executed, and thus focus mostly on implementation and less on representation of sequence skill. Though this endeavor probably results in an oversimplification and a somewhat artificial separation of massively interacting networks (e.g., cortico-striatal loops cannot be strictly separated; Seger and Spiering, 2011), we believe that this effort will guide future research. In brief, we propose that S-R based performance in the reaction mode is related to the associative cortico-striatal loop (AL) in concert with prefrontal cortex (AL_PFC_). With practice, sensorimotor cortico-striatal loops (SLs) gradually take over and enable both more automatic S-R translation and sequence based performance in close interaction with premotor and primary motor cortices^3^. For the associative mode we propose the sensorimotor loop to involve the premotor cortex (SL_PMC_), while for the chunking mode the SMA is involved instead (SL_SMA_). In the chunking mode, an AL_PRE−SMA_ loop may remain involved for the actual loading of motor chunks. Hence, besides building from the accepted distinction between the AL and the SL, we also propose functional divisions of both the AL and the SL.

### Reaction mode

The execution of an individual movement on the basis of an external stimulus (like when a random or unfamiliar sequence is being executed) probably involves areas that are consistently related to spatial response selection, such as the premotor cortex (PMC), the parietal cortex and the prefrontal cortex (PFC) (Iacoboni et al., 1996; Dassonville et al., 2001; Merriam et al., 2001; Schumacher and D'Esposito, 2002; Jiang and Kanwisher, 2003; Schumacher et al., 2003, 2005, 2007). The associative striatum enables a functional network between prefrontal and posterior areas (i.e., AL; Seger, 2008) to support the initial S-R translation processes that underlie the reaction mode (i.e., performance is driven by goal-directed control based on the S-R mappings that are held in working memory). Indeed, activity in the associative striatum has been linked to the early stages of training in sequence learning and habit formation tasks (Jankowski et al., 2009; Ashby et al., 2010). Moreover, it has been shown that activity in the associative striatum (i.e., anterior caudate) is closely correlated with (the rate of) learning the associations between visual cues and specific motor responses (Williams and Eskandar, 2006). However, the involvement of PFC may soon decrease as the highly compatible spatial S-R mapping of the DSP task allows for less controlled response selection that involves PMC in concert with the sensorimotor striatum—in line with the special role that is assumed for PMC in translating spatial information into motor output (Hikosaka et al., 1999) and with PMC involvement in habit formation (i.e., automatic S-R translation; Ashby et al., 2010).

### Sequence skill

With more practice and the development of a sequence representation, activity will further shift from the AL toward SLs. The SLs are networks that involve the sensorimotor striatum, premotor (PMC, supplementary motor area or SMA) and motor cortices. Various findings support this notion of activity shifts. First, Miyachi et al. (2002) found that the sensorimotor striatum is home to most of the striatal neurons that show their strongest response to highly practiced motor sequences. Furthermore, whereas the temporary inactivation of the sensorimotor striatum impairs performance on already acquired motor sequences, it hardly affects the learning of new motor sequences (Miyachi et al., 1997). Second, practice-based transition in activity can also be observed at the cortical level. Specifically, whereas PMC is typically activated relatively early in learning, later in training this activation decreases while SMA activity gradually increases (Jenkins et al., 1994; Toni et al., 1998; Wymbs and Grafton, 2013). It is assumed that SMA is strongly related to memory-based sequence performance (Mushiake et al., 1991; Haaland et al., 2004), thus independent of external action cuing, while PMC underlies skill that is stimulus-based. Below we specify this for both the associative and chunking modes that we defined above, starting with the latter because it better relates to the existing neuro-imaging work with discrete movement sequences.

#### Chunking mode

The crucial role of the BG for motor chunking has become evident over the last decades. Studies on stroke (Boyd et al., 2009) and Parkinson's disease (e.g., Hayes et al., 1998; Tremblay et al., 2010) led to the conclusion that the ability to form motor chunks is impaired in patients with BG damage. Additionally, rodent research has shown that activity in the striatum is strongly related to, and essential for, motor chunking (Yin and Knowlton, 2006; Graybiel, 2008; Jin and Costa, 2010). Performance in the chunking mode is dominated by the cognitive processor selecting and loading a motor chunk that is subsequently executed by the motor processor. While the overall involvement of BG is evident, we here speculate about the chunking mode in some more detail, subsequently considering (1) the segmentation of sequences, (2) the motor buffer, (3) the loading of the motor buffer, and (4) chunk-based performance.

First, as noted before, discrete movement sequences exceeding about four or five responses are usually spontaneously segmented into two parts. Recent studies suggest that such segmentation of longer sequences into multiple smaller chunks is based on fronto-parietal networks. Pammi et al. (2012) observed selective activation of a fronto-parietal network in the early learning stage with increasing sequence length (in the *m* × *n* task). This notion also fits well with two studies by Verwey and colleagues who showed that the ability to segment long sequences into chunks is impaired in elderly (Verwey, 2010; Verwey et al., 2011), which could be related to reduced cortical capacity (Resnick et al., 2003; Raz et al., 2005). The segments that are created can be assumed to gradually transform into relatively rigid motor chunks, with concatenation processes required for the fluid transitions between motor chunks. In a recent fMRI study on human subjects, Wymbs et al. (2012) related these latter processes to the bilateral putamen of the BG.

Second, the chunking mode involves reading responses from a motor buffer. As noted above, we conceive of the motor buffer as a part of working memory. Over the last decades, an increasing number of researchers understand working memory as the activated part of long term memory (e.g., Cowan, 1995; Postle, 2006). The long term representations for sequence skill (i.e., motor chunks) are highly distributed, and may even shift between areas with practice. However, there is no overall consensus. For example, storage has been proposed to relate to premotor areas (Jacobsen, 1934; Fulton, 1935), to the sensorimotor parts of the BG (Lehéricy et al., 2005), to the cerebellum (e.g., Hikosaka et al., 2002; Doyon et al., 2009), and, with long term practice, to the primary motor cortex itself (e.g., Matsuzaka et al., 2007). Additionally, equally strong arguments have been proposed against some candidate regions. For example, PMC activation may not reflect the representation of motor commands *per se* but rather their associations with specific sensory cues (e.g., Halsband and Lange, 2006), while the BG may contribute to skill by training cortical-cortical and thalamo-cortical representations rather than by storing procedural knowledge (e.g., Ashby et al., 2010; Desmurget and Turner, 2010). Overall, then, it is difficult to pinpoint the representation that develops with short, discrete keying sequences in the DSP task. Sequence representations are probably highly task- and context-dependent, and relevant neuro-imaging work with the DSP task is currently lacking.

Third, on the basis of a study by Kennerley et al. (2004) we propose that loading the motor buffer (in the chunking mode) is related to pre-SMA. In this TMS study the authors showed for extensively practiced sequences (a) that the pre-SMA is involved in the initiation of a motor chunk, but (b) that this only holds when the motor chunk needs to be retrieved from memory as a “superordinate set of movements without the aid of a visuomotor association” (p. 978). Conversely, the pre-SMA was shown to not be involved in general execution processes. Pre-SMA, then, through its dense connections with PFC, is assumed here to selectively activate the relevant long-term memory representations (i.e., load the motor buffer) that are stored elsewhere. This initiating role of the pre-SMA fits well with findings from monkey research that pre-SMA neurons are mostly active during pre-movement and not during actual movement (Halsband and Lange, 2006). Because pre-SMA is typically related to the AL with the basal ganglia, the loading of the motor buffer may require a stable involvement of the AL_pre−SMA_ in even more advanced sequence skill, although, as mentioned above, the AL_PFC_ gradually reduces its impact.

Fourth, the true chunking based performance is proposed to rely on the SL_SMA_. This fits well with the notion that SMA is typically involved in memory-based performance: though stimuli are still presented in the DSP task even after substantial practice, these are assumed to be no longer dominant in the response selection process—as evidenced, among others, by average RTs of sometimes below 100 ms. It is also consistent with various other findings. For example, a study with mice by Jin and Costa (2010) indicates that initiating (and also aborting) action sequences is related to nigro-striatal circuits—as if start (and stop) signals are represented within these circuits. In sum, from the notion that action sequences are generally goal-directed, we propose that initiation of well-learned action sequences is based on sequence (or motor chunk) selection and loading through PFC (Averbeck et al., 2006) and pre-SMA, after which a sequence-specific SL_SMA_ is involved in prompting sequence execution.

Finally, we could speculate on a different (or possibly just complementary) function for the BG in sequence skill. Specifically, as discrete sequence skill involves the activation by PFC/pre-SMA of particular sequence (motor chunk) representations laid out somewhere else in the brain (i.e., loading the motor buffer; see above), the effectiveness of this advance preparation can be assumed to require the temporary inhibition of execution processes. The BG are well-suited to moderate this process as they are involved in go- (cf. direct pathway) and no-go-signals (cf. indirect and hyperdirect pathways; Nambu et al., 2002) that determine thalamico-cortical output. Various observations are in line with such a moderator role. For example, the BG have been shown to be heavily involved in tasks that require inhibiting a planned action program such as in the stop-signal task (Aron and Poldrack, 2006), and there is at least tentative support for BG involvement in motor imagery (Guillot et al., 2012), which probably also relates to the inhibition of motor commands. Moreover, Elsinger et al. (2006) observed enhanced activity in the anterior putamen when sequences were held in memory for delayed execution, which could be related to inhibitory processes as well. As such, loading of the motor buffer during the preparation of skilled DSP may require inhibitory processes within BG.

#### Associative mode

We propose that the major difference between the chunking and the associative mode relates to the sensorimotor loop that is involved. Whereas the SL_SMA_ loop underlies the chunking mode, the associative mode builds from a SL_PMC_ because performance in the associative mode is still partly under stimulus-based control. The latter loop will be engaged either when practice has not yet developed strong enough representations for memory-based performance (i.e., the chunking mode driven by the SL_SMA_), or when the chunking mode has been disengaged through experimental manipulations. This fits well with studies that relate both the SL and the PMC to implicit sequence learning in the SRT task (e.g., Grafton et al., 2002; Bischoff-Grethe et al., 2004; Seger, 2006), which is typically seen as a form of associative learning (e.g., Abrahamse et al., 2010) that remains at least partly stimulus-driven and does not include motor chunking (Jiménez et al., 2011). Also inspired by the SRT literature, the storage in the brain of knowledge that underlies the associative mode is highly task- and/or context-dependent, but probably involves at least areas across parietal cortex (e.g., Jenkins et al., 1994; Grafton et al., 1998) that are related to visuo-spatial coding.

## Conclusions and questions for future research

In the current paper we have described the DSP task, the major behavioral phenomena that can be typically observed with it, and an update of the DPM. The DPM holds that discrete sequence skill builds from the continuous and dynamic interplay between a cognitive processor and a motor system comprising a motor processor and a motor buffer, with the former being dominant early on in practice, and the latter taking over execution as practice evolves. The notion that movement skill is characterized by automaticity is explained by the relative autonomy of the motor system from the cognitive processor. As we have outlined, this model generates various predictions of the model at the behavioral level that await further exploration. We have emphasized that the DSP literature that underlies the DPM is limited in scope in terms of practice amount and sequence structure, and future studies should aim to clarify how the DPM relates to these features; from there is should also be explored if the general notions of DPM hold across other sequence learning paradigms.

As to the neural underpinnings of the DPM, we suggest (a) that striatum and PMC (possibly in concert with more posterior areas) define a functional loop that underlies the reaction mode from the moment that S-R translation becomes relatively automatic (cf. habit formation). In the case of the DSP task this would develop quite rapidly because of the high spatial compatibility of stimuli and responses. We further suggest (b) that a sensorimotor-PMC loop underlies the associative mode, and (c) that a sensorimotor-SMA loop underlies the chunking mode. The main distinction between the associative and the chunking modes may lie in the efforts of the BG to inhibit execution during the activation of (cortical or subcortical) areas that contain relevant sequence representations. Besides generating predictions for future research, we believe that this tentative mapping of the DPM's execution modes on specific cortico-striatal loops will contribute to explorations on the biological plausibility of DPM.

### Conflict of interest statement

The authors declare that the research was conducted in the absence of any commercial or financial relationships that could be construed as a potential conflict of interest.

## Footnotes

^1^Motor adaptation, a form of (re-)learning characterized by gradual improvement in performance in response to altered task conditions, can be studied both with arm and eye movements, and either by using visuomotor adaptation (i.e., distortion of the visual but not the proprioceptive consequences of the motor commands) or by using force-field adaptation (i.e., distortion of both the visual and proprioceptive consequences of motor commands). For reviews see Krakauer and Mazzoni (2011) and Shadmehr et al. (2010).

^2^See this link for a downloadable EPrime version of the DSP task: http://www.utwente.nl/gw/cpe/en/Employees%20CPE/Verwey/Research/research.doc/

^3^It should be noted that, with an amount of practice that exceeds the level typical for the DSP task, the sensorimotor cortico-striatal loops may even enable direct cortical-cortical representations to form on the base of slow Hebbian learning (Ashby et al., 2010; Karni et al., 1998). Yet, this will not be covered in the present review.
